# 4-Methyl-2,4,6-triphenyl-4*H*-thio­pyran

**DOI:** 10.1107/S1600536809005911

**Published:** 2009-02-25

**Authors:** Hossein Rahmani, Hooshang Pirelahi, Seik Weng Ng

**Affiliations:** a Institute of Chemical Industries, Iranian Research Organization for Science and Technology, PO Box 15815-358, Tehran, Iran; b Department of Chemistry, College of Science, University of Tehran, PO Box 13145-143, Tehran, Iran; c Department of Chemistry, University of Malaya, 50603 Kuala Lumpur, Malaysia

## Abstract

The six-membered thio­pyran ring in the title compound, C_24_H_20_S, adopts a flattened boat conformation, with the S atom displaced by 0.273 (2) Å and the 3-methyl­ene C atom by 0.294 (3) Å from the plane of the other four *sp* ^2^-hydridized C atoms. The methyl group on the methyl­ene carbon lies in a axial position with the phenyl equatorial.

## Related literature

2,4,4,6-Tetraaryl- or 4-alkyl-2,4,6-triaryl-4*H*-thio­pyrans undergo UV-induced isomerization to form aryl-migrated 2*H*-thio­pyrans; for a discussion of the photoisomerization mechanism, see: Pirelahi *et al.* (2004 ▶); Pirelahi & Rahmani (1997 ▶). 4-Methyl-2,4,6-triphenyl-4*H*-thio­pyran does not react in the solid state, but in solution is converted to 4-methyl-2,3,6-triphenyl-2*H*-thio­pyran; see: Mori & Maeda (1991 ▶). For the synthesis, see: Suld & Price (1962 ▶).
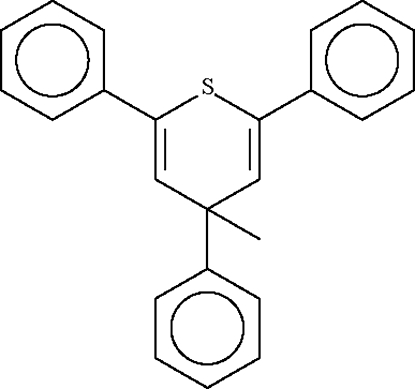


## Experimental

### 

#### Crystal data

C_24_H_20_S
*M* *_r_* = 340.46
Monoclinic, 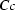

*a* = 9.8737 (2) Å
*b* = 22.5282 (4) Å
*c* = 9.2288 (2) Å
β = 118.987 (1)°
*V* = 1795.67 (6) Å^3^
*Z* = 4
Mo *K*α radiation
μ = 0.18 mm^−1^
*T* = 115 K
0.35 × 0.20 × 0.10 mm

#### Data collection

Bruker SMART APEX diffractometer
Absorption correction: multi-scan (*SADABS*; Sheldrick, 1996 ▶) *T*_min_ = 0.917, *T*_max_ = 0.982
8464 measured reflections
3883 independent reflections
3698 reflections with *I* > σ(*I*)
*R*_int_ = 0.024

#### Refinement

*R*[*F* ^2^ > 2σ(*F* ^2^)] = 0.029
*wR*(*F* ^2^) = 0.072
*S* = 1.03
3883 reflections
227 parameters
2 restraints
H-atom parameters constrained
Δρ_max_ = 0.28 e Å^−3^
Δρ_min_ = −0.18 e Å^−3^
Absolute structure: Flack (1983 ▶), 1825 Friedel pairs
Flack parameter: 0.00 (5)

### 

Data collection: *APEX2* (Bruker, 2008 ▶); cell refinement: *SAINT* (Bruker, 2008 ▶); data reduction: *SAINT*; program(s) used to solve structure: *SHELXS97* (Sheldrick, 2008 ▶); program(s) used to refine structure: *SHELXL97* (Sheldrick, 2008 ▶); molecular graphics: *X-SEED* (Barbour, 2001 ▶); software used to prepare material for publication: *publCIF* (Westrip, 2009 ▶).

## Supplementary Material

Crystal structure: contains datablocks global, I. DOI: 10.1107/S1600536809005911/sj2578sup1.cif

Structure factors: contains datablocks I. DOI: 10.1107/S1600536809005911/sj2578Isup2.hkl

Additional supplementary materials:  crystallographic information; 3D view; checkCIF report

## Figures and Tables

**Fig. 1.**
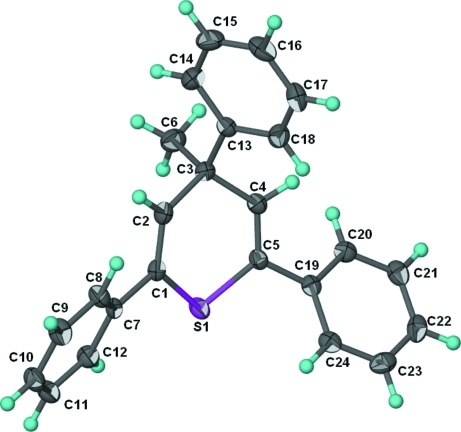
Thermal ellisoid plot (Barbour, 2001) of C24H20S; probability levels are set at 70% and H-atoms are drawn as spheres of arbitrary radius.

## supplementary crystallographic information

### Experimental

The compound was synthesized by the reaction of methyl magnesium bromide and 2,4,6-triphenylthiopyrylium perchlorate in dry ether under an argon atmosphere according to a reported method (Suld & Price, 1962). The product was isolated by TLC on neutral alumina (petroleum ether 40–60 °C) and purified by recrystalization from ethanol.

### Refinement

Carbon-bound H-atoms were placed in calculated positions (C–H 0.95 to 0.98 Å) and were included in the refinement in the riding model approximation, with *U*(H) set to 1.2 to 1.5*U*(C).

### Crystal data

**Table:**

C_24_H_20_S	*F*(000) = 720
*M**_r_* = 340.46	*D*_x_ = 1.259 Mg m^−^^3^
Monoclinic, *C**c*	Mo *K*α radiation, λ = 0.71073 Å
Hall symbol: C -2yc	Cell parameters from 4067 reflections
*a* = 9.8737 (2) Å	θ = 2.5–28.0°
*b* = 22.5282 (4) Å	µ = 0.18 mm^−^^1^
*c* = 9.2288 (2) Å	*T* = 115 K
β = 118.987 (1)°	Prism, colorless
*V* = 1795.67 (6) Å^3^	0.35 × 0.20 × 0.10 mm
*Z* = 4	

### Data collection

**Table:**

Bruker SMART APEX diffractometer	3883 independent reflections
Radiation source: fine-focus sealed tube	3698 reflections with *I* > σ(*I*)
graphite	*R*_int_ = 0.024
ω scans	θ_max_ = 27.5°, θ_min_ = 1.8°
Absorption correction: multi-scan (*SADABS*; Sheldrick, 1996)	*h* = −12→12
*T*_min_ = 0.917, *T*_max_ = 0.982	*k* = −29→29
8464 measured reflections	*l* = −11→11

### Refinement

**Table:**

Refinement on *F*^2^	Secondary atom site location: difference Fourier map
Least-squares matrix: full	Hydrogen site location: inferred from neighbouring sites
*R*[*F*^2^ > 2σ(*F*^2^)] = 0.029	H-atom parameters constrained
*wR*(*F*^2^) = 0.072	*w* = 1/[σ^2^(*F*_o_^2^) + (0.0401*P*)^2^ + 0.2571*P*] where *P* = (*F*_o_^2^ + 2*F*_c_^2^)/3
*S* = 1.03	(Δ/σ)_max_ = 0.001
3883 reflections	Δρ_max_ = 0.28 e Å^−^^3^
227 parameters	Δρ_min_ = −0.17 e Å^−^^3^
2 restraints	Absolute structure: Flack (1983), 1825 Friedel pairs
Primary atom site location: structure-invariant direct methods	Flack parameter: 0.00 (5)

### Fractional atomic coordinates and isotropic or equivalent isotropic displacement parameters (Å^2^)

**Table:**

	*x*	*y*	*z*	*U*_iso_*/*U*_eq_	
S1	0.49977 (4)	0.892322 (17)	0.49963 (4)	0.01993 (9)	
C1	0.48277 (17)	0.87133 (6)	0.67575 (18)	0.0169 (3)	
C2	0.37550 (18)	0.83350 (7)	0.66660 (19)	0.0197 (3)	
H2	0.3740	0.8259	0.7671	0.024*	
C3	0.25547 (17)	0.80107 (7)	0.51476 (19)	0.0187 (3)	
C4	0.22225 (18)	0.83423 (7)	0.35828 (18)	0.0180 (3)	
H4	0.1228	0.8283	0.2651	0.022*	
C5	0.31709 (16)	0.87086 (6)	0.33675 (17)	0.0156 (3)	
C6	0.3178 (2)	0.73921 (7)	0.5054 (2)	0.0252 (4)	
H6A	0.3487	0.7178	0.6092	0.038*	
H6B	0.4075	0.7437	0.4879	0.038*	
H6C	0.2366	0.7168	0.4131	0.038*	
C7	0.59735 (17)	0.90088 (7)	0.83149 (19)	0.0174 (3)	
C8	0.55816 (18)	0.91435 (7)	0.95401 (19)	0.0210 (3)	
H8	0.4585	0.9041	0.9379	0.025*	
C9	0.66275 (19)	0.94253 (7)	1.0990 (2)	0.0241 (3)	
H9	0.6341	0.9517	1.1810	0.029*	
C10	0.8091 (2)	0.95738 (7)	1.1249 (2)	0.0236 (4)	
H10	0.8809	0.9766	1.2245	0.028*	
C11	0.84996 (18)	0.94407 (7)	1.0045 (2)	0.0237 (3)	
H11	0.9503	0.9540	1.0221	0.028*	
C12	0.74509 (18)	0.91643 (7)	0.85877 (19)	0.0207 (3)	
H12	0.7738	0.9079	0.7765	0.025*	
C13	0.10640 (17)	0.79667 (7)	0.52855 (18)	0.0183 (3)	
C14	0.0894 (2)	0.75238 (8)	0.6233 (2)	0.0255 (3)	
H14	0.1679	0.7231	0.6742	0.031*	
C15	−0.0402 (2)	0.75009 (8)	0.6451 (2)	0.0294 (4)	
H15	−0.0487	0.7198	0.7118	0.035*	
C16	−0.1572 (2)	0.79169 (8)	0.5702 (2)	0.0284 (4)	
H16	−0.2465	0.7899	0.5836	0.034*	
C17	−0.14174 (19)	0.83609 (8)	0.47516 (19)	0.0257 (4)	
H17	−0.2212	0.8649	0.4232	0.031*	
C18	−0.01103 (19)	0.83879 (7)	0.45533 (18)	0.0222 (3)	
H18	−0.0016	0.8698	0.3910	0.027*	
C19	0.27339 (17)	0.89872 (7)	0.17441 (18)	0.0165 (3)	
C20	0.18705 (18)	0.86626 (7)	0.02926 (18)	0.0191 (3)	
H20	0.1658	0.8255	0.0359	0.023*	
C21	0.13179 (19)	0.89302 (7)	−0.1250 (2)	0.0222 (3)	
H21	0.0729	0.8706	−0.2230	0.027*	
C22	0.16242 (19)	0.95233 (8)	−0.1358 (2)	0.0238 (4)	
H22	0.1232	0.9708	−0.2412	0.029*	
C23	0.25068 (19)	0.98482 (7)	0.0078 (2)	0.0242 (3)	
H23	0.2722	1.0255	0.0005	0.029*	
C24	0.30752 (18)	0.95805 (7)	0.16187 (18)	0.0192 (3)	
H24	0.3700	0.9802	0.2594	0.023*	

### Atomic displacement parameters (Å^2^)

**Table:**

	*U*^11^	*U*^22^	*U*^33^	*U*^12^	*U*^13^	*U*^23^
S1	0.01477 (16)	0.02749 (19)	0.01650 (17)	−0.00309 (16)	0.00676 (13)	−0.00013 (15)
C1	0.0159 (7)	0.0177 (7)	0.0151 (7)	0.0022 (6)	0.0060 (6)	0.0015 (6)
C2	0.0202 (7)	0.0213 (8)	0.0151 (7)	0.0008 (6)	0.0067 (6)	0.0030 (6)
C3	0.0183 (7)	0.0174 (7)	0.0179 (7)	−0.0021 (6)	0.0069 (6)	0.0014 (5)
C4	0.0177 (7)	0.0189 (7)	0.0146 (7)	−0.0021 (6)	0.0056 (6)	−0.0022 (6)
C5	0.0144 (7)	0.0154 (7)	0.0151 (7)	0.0006 (5)	0.0056 (6)	−0.0027 (5)
C6	0.0260 (9)	0.0207 (8)	0.0305 (9)	0.0010 (7)	0.0150 (7)	0.0016 (7)
C7	0.0144 (7)	0.0168 (7)	0.0159 (7)	0.0014 (6)	0.0032 (6)	0.0022 (5)
C8	0.0149 (7)	0.0237 (8)	0.0219 (8)	−0.0006 (6)	0.0070 (6)	−0.0016 (6)
C9	0.0233 (8)	0.0294 (8)	0.0197 (8)	0.0019 (7)	0.0105 (6)	−0.0021 (6)
C10	0.0203 (8)	0.0216 (8)	0.0206 (8)	−0.0008 (7)	0.0034 (6)	−0.0044 (6)
C11	0.0167 (7)	0.0261 (8)	0.0250 (8)	−0.0027 (6)	0.0075 (6)	−0.0014 (6)
C12	0.0201 (8)	0.0237 (8)	0.0190 (8)	−0.0008 (6)	0.0100 (6)	0.0002 (6)
C13	0.0172 (7)	0.0187 (7)	0.0153 (7)	−0.0034 (6)	0.0048 (6)	−0.0022 (5)
C14	0.0258 (8)	0.0221 (8)	0.0262 (8)	−0.0006 (7)	0.0107 (7)	0.0051 (6)
C15	0.0318 (9)	0.0282 (9)	0.0302 (9)	−0.0090 (7)	0.0167 (8)	0.0027 (7)
C16	0.0208 (8)	0.0378 (10)	0.0267 (9)	−0.0079 (7)	0.0115 (7)	−0.0065 (7)
C17	0.0198 (8)	0.0340 (9)	0.0182 (8)	0.0035 (7)	0.0050 (6)	−0.0011 (6)
C18	0.0246 (8)	0.0228 (8)	0.0166 (8)	−0.0005 (6)	0.0079 (7)	0.0008 (5)
C19	0.0140 (7)	0.0203 (7)	0.0165 (8)	0.0010 (6)	0.0084 (6)	−0.0008 (6)
C20	0.0188 (7)	0.0198 (7)	0.0202 (8)	−0.0007 (6)	0.0106 (6)	−0.0012 (6)
C21	0.0184 (8)	0.0318 (9)	0.0166 (8)	−0.0006 (6)	0.0086 (6)	−0.0019 (6)
C22	0.0195 (8)	0.0346 (9)	0.0197 (8)	0.0068 (7)	0.0113 (7)	0.0079 (7)
C23	0.0262 (8)	0.0216 (8)	0.0314 (9)	−0.0006 (7)	0.0191 (7)	0.0027 (6)
C24	0.0182 (7)	0.0222 (8)	0.0192 (8)	−0.0019 (6)	0.0106 (6)	−0.0027 (6)

### Geometric parameters (Å, °)

**Table:**

S1—C5	1.7661 (14)	C11—H11	0.9500
S1—C1	1.7772 (15)	C12—H12	0.9500
C1—C2	1.330 (2)	C13—C14	1.390 (2)
C1—C7	1.488 (2)	C13—C18	1.393 (2)
C2—C3	1.514 (2)	C14—C15	1.390 (2)
C2—H2	0.9500	C14—H14	0.9500
C3—C4	1.514 (2)	C15—C16	1.384 (3)
C3—C13	1.541 (2)	C15—H15	0.9500
C3—C6	1.543 (2)	C16—C17	1.386 (3)
C4—C5	1.333 (2)	C16—H16	0.9500
C4—H4	0.9500	C17—C18	1.390 (2)
C5—C19	1.483 (2)	C17—H17	0.9500
C6—H6A	0.9800	C18—H18	0.9500
C6—H6B	0.9800	C19—C20	1.396 (2)
C6—H6C	0.9800	C19—C24	1.397 (2)
C7—C8	1.394 (2)	C20—C21	1.391 (2)
C7—C12	1.399 (2)	C20—H20	0.9500
C8—C9	1.386 (2)	C21—C22	1.384 (2)
C8—H8	0.9500	C21—H21	0.9500
C9—C10	1.387 (3)	C22—C23	1.390 (2)
C9—H9	0.9500	C22—H22	0.9500
C10—C11	1.386 (2)	C23—C24	1.388 (2)
C10—H10	0.9500	C23—H23	0.9500
C11—C12	1.386 (2)	C24—H24	0.9500
			
C5—S1—C1	101.27 (7)	C11—C12—C7	120.71 (15)
C2—C1—C7	123.89 (14)	C11—C12—H12	119.6
C2—C1—S1	122.53 (12)	C7—C12—H12	119.6
C7—C1—S1	113.57 (11)	C14—C13—C18	117.92 (14)
C1—C2—C3	127.59 (14)	C14—C13—C3	120.65 (14)
C1—C2—H2	116.2	C18—C13—C3	121.32 (14)
C3—C2—H2	116.2	C15—C14—C13	121.22 (16)
C4—C3—C2	110.82 (12)	C15—C14—H14	119.4
C4—C3—C13	109.66 (12)	C13—C14—H14	119.4
C2—C3—C13	107.77 (13)	C16—C15—C14	120.43 (16)
C4—C3—C6	107.47 (13)	C16—C15—H15	119.8
C2—C3—C6	109.47 (13)	C14—C15—H15	119.8
C13—C3—C6	111.67 (12)	C15—C16—C17	118.94 (16)
C5—C4—C3	127.18 (13)	C15—C16—H16	120.5
C5—C4—H4	116.4	C17—C16—H16	120.5
C3—C4—H4	116.4	C16—C17—C18	120.56 (16)
C4—C5—C19	122.09 (13)	C16—C17—H17	119.7
C4—C5—S1	122.96 (11)	C18—C17—H17	119.7
C19—C5—S1	114.91 (11)	C17—C18—C13	120.92 (15)
C3—C6—H6A	109.5	C17—C18—H18	119.5
C3—C6—H6B	109.5	C13—C18—H18	119.5
H6A—C6—H6B	109.5	C20—C19—C24	118.78 (14)
C3—C6—H6C	109.5	C20—C19—C5	119.41 (13)
H6A—C6—H6C	109.5	C24—C19—C5	121.68 (13)
H6B—C6—H6C	109.5	C21—C20—C19	120.60 (15)
C8—C7—C12	118.32 (14)	C21—C20—H20	119.7
C8—C7—C1	120.06 (14)	C19—C20—H20	119.7
C12—C7—C1	121.62 (14)	C22—C21—C20	120.09 (15)
C9—C8—C7	120.86 (15)	C22—C21—H21	120.0
C9—C8—H8	119.6	C20—C21—H21	120.0
C7—C8—H8	119.6	C21—C22—C23	119.82 (15)
C8—C9—C10	120.22 (15)	C21—C22—H22	120.1
C8—C9—H9	119.9	C23—C22—H22	120.1
C10—C9—H9	119.9	C24—C23—C22	120.22 (15)
C11—C10—C9	119.59 (15)	C24—C23—H23	119.9
C11—C10—H10	120.2	C22—C23—H23	119.9
C9—C10—H10	120.2	C23—C24—C19	120.44 (14)
C12—C11—C10	120.30 (15)	C23—C24—H24	119.8
C12—C11—H11	119.9	C19—C24—H24	119.8
C10—C11—H11	119.9		
			
C5—S1—C1—C2	17.61 (15)	C4—C3—C13—C14	157.18 (14)
C5—S1—C1—C7	−161.33 (11)	C2—C3—C13—C14	−82.11 (17)
C7—C1—C2—C3	−179.48 (15)	C6—C3—C13—C14	38.2 (2)
S1—C1—C2—C3	1.7 (2)	C4—C3—C13—C18	−26.78 (19)
C1—C2—C3—C4	−24.4 (2)	C2—C3—C13—C18	93.93 (16)
C1—C2—C3—C13	−144.35 (16)	C6—C3—C13—C18	−145.80 (14)
C1—C2—C3—C6	94.01 (19)	C18—C13—C14—C15	−0.2 (2)
C2—C3—C4—C5	26.5 (2)	C3—C13—C14—C15	175.99 (16)
C13—C3—C4—C5	145.30 (15)	C13—C14—C15—C16	1.0 (3)
C6—C3—C4—C5	−93.12 (19)	C14—C15—C16—C17	−0.9 (3)
C3—C4—C5—C19	177.18 (14)	C15—C16—C17—C18	0.0 (3)
C3—C4—C5—S1	−5.5 (2)	C16—C17—C18—C13	0.8 (2)
C1—S1—C5—C4	−15.81 (15)	C14—C13—C18—C17	−0.7 (2)
C1—S1—C5—C19	161.67 (11)	C3—C13—C18—C17	−176.85 (14)
C2—C1—C7—C8	−31.3 (2)	C4—C5—C19—C20	−37.1 (2)
S1—C1—C7—C8	147.59 (12)	S1—C5—C19—C20	145.37 (12)
C2—C1—C7—C12	149.37 (16)	C4—C5—C19—C24	138.58 (16)
S1—C1—C7—C12	−31.71 (19)	S1—C5—C19—C24	−38.92 (19)
C12—C7—C8—C9	0.1 (2)	C24—C19—C20—C21	−2.1 (2)
C1—C7—C8—C9	−179.21 (15)	C5—C19—C20—C21	173.78 (14)
C7—C8—C9—C10	−0.5 (3)	C19—C20—C21—C22	0.1 (2)
C8—C9—C10—C11	0.2 (3)	C20—C21—C22—C23	1.0 (2)
C9—C10—C11—C12	0.4 (3)	C21—C22—C23—C24	−0.3 (2)
C10—C11—C12—C7	−0.8 (3)	C22—C23—C24—C19	−1.7 (2)
C8—C7—C12—C11	0.5 (2)	C20—C19—C24—C23	2.8 (2)
C1—C7—C12—C11	179.82 (15)	C5—C19—C24—C23	−172.93 (14)
